# Student Loans and Psychological Distress: A Cross-sectional Study of Young Adults in Japan

**DOI:** 10.2188/jea.JE20190057

**Published:** 2020-10-05

**Authors:** Yukihiro Sato, Richard G. Watt, Yasuaki Saijo, Eiji Yoshioka, Ken Osaka

**Affiliations:** 1 Division of Public Health and Epidemiology, Department of Social Medicine, Asahikawa Medical University, Asahikawa, Japan; 2 Department of Epidemiology and Public Health, University College London, London, United Kingdom; 3 Department of International and Community Oral Health, Tohoku University Graduate School of Dentistry, Sendai, Japan

**Keywords:** mental health, student health, student loans

## Abstract

**Background:**

Levels of student loan debt have been increasing, but very little research has assessed if this is associated with poor health. The aim was to examine the association between student loans and psychological distress in Japan.

**Methods:**

We conducted a cross-sectional web-based self-administered questionnaire survey in 2017. The sample comprised of 4,149 respondents aged 20–34, with 3,170 graduates and 979 current university students. The independent variables were whether or not current students had student loans, and for graduates, the total amount of their student loan debt. The dependent variable was severe psychological distress assessed using the Kessler Psychological Distress Scale (K6; the cut-off point was 12/13). Covariates were demographic and parents’ socioeconomic variables. A Poisson regression analysis with a robust error variance was conducted to estimate prevalence ratios (PRs) and 95% confidence intervals (CIs). Because there was a significant interaction between current student status and the status of borrowing student loans, stratified analyses were conducted.

**Results:**

The percentage of those with student loans was 33.8% among graduates and 35.2% among current university students. Among graduates, student loan debt was significantly associated with a high possibility of having severe psychological distress after adjusting for covariates (PR of ≥4 million yen, 1.44; 95% CI, 1.02–2.03). Among current university students, there was no significant association (PR of borrowing student loans, 0.91; 95% CI, 0.60–1.37).

**Conclusions:**

There was a significant association between student loan debt and psychological distress among graduates but not current university students.

## INTRODUCTION

Levels of debt have been associated with physical and mental health problems.^1^^,^^2^ Recent reviews show the significant associations of household debt with mental disorder and depression,^3^^–^^5^ because debt repayment can directly lead to financial stress, anxiety, unhealthy behaviors,^2^^,^^6^ and limitations of using healthcare services.^7^ Earlier studies also indicate the negative effects of debt on mental health among young adults.^3^^,^^8^ The amount of household debt has been increasing worldwide,^9^ and student loans have contributed to the rise in personal debt.^3^^,^^8^

The annual amount of student loan debt has been exponentially increasing due to increases in tuition fees and increases in private expenditure in tertiary education.^10^ In the United Kingdom and the United States, 92% and 62% of students, respectively, benefit from student loans.^11^^,^^12^ Nowadays, student loans have become a widely accepted approach for low- and middle-income households to facilitate educational attainment, especially across the Organisation for Economic Co-operation and Development (OECD) countries.^12^

Few studies have reported the associations between student loans and health. Walsemann and colleagues reported that borrowing student loans was associated with worse mental health^13^ and shorter sleep duration among young adults in the United States.^14^ Other studies also indicated the negative impacts of student loans on psychological health and access to healthcare services.^7^^,^^15^ However, to our knowledge, no study focuses on the associations in Japan.

In Japan, student loans for tertiary education have been increasing, because scholarships are not widely available compared with other OECD countries.^11^^,^^16^ Indeed, there were no scholarships funded by the Japanese government and incorporated administrative agencies until 2018.^11^^,^^16^ Scholarships are mainly provided by private sector organizations; however, they are scarce.^11^^,^^16^^,^^17^ The Japan Student Services Organization (JASSO) is a quasi-governmental agency, and they are the main provider of student loans in Japan.^16^^,^^17^ Earlier reports estimated that among all of the university students in Japan, 27.2 to 42.3% of them borrowed student loans from the JASSO,^17^^,^^18^ and only 1.8% of students had student loans from other funds.^18^ Students with scholarships only were 2.0–6.3%, and students with both student loans and scholarships were 0.3–1.7%.^17^^,^^18^ Low- and middle-income households in Japan have borrowed student loans.

JASSO provides two types of student loans.^16^ Type 1 are student loans without interest, and type 2 are loans with interest, but it is relatively low.^16^ The typical mean amounts of type 1 and type 2 student loan debt are estimated at 2.36 and 3.43 million yen (approximately equivalent to 23,600 USD and 34,300 USD) per student, respectively.^16^ JASSO demands monthly repayment starting 7 months after graduation for periods of 14 to 20 years.^16^ The typical amounts of monthly repayment range from 11,293 to 32,297 yen, depending on the amount and the types of student loans.^16^ Although the mean monthly initial income of graduated adults has almost unchanged from 200,000 yen for over a decade,^19^ the amount of student loans has been increasing rapidly.^16^

Considering that current university students obtain monetary support from student loans, it is plausible that the associations between student loans and psychological distress are weak among students. On the other hands, after graduation or dropout, the cumulative amount of student loan debt can be a burden; therefore, the associations might be strengthened among graduates and dropouts. We hypothesized that, while there is no association between student loans and psychological distress among current university students, student loan debt is associated with psychological distress among graduates and dropouts. The aim of this cross-sectional study was to examine whether borrowing student loans and the total amount of student loan debt were associated with psychological distress among graduates and dropouts in Japan. Besides, we also examined whether borrowing student loans was associated with psychological distress among current university students.

## METHODS

### Study design, data sources, and participants

This was a cross-sectional study. The study population was obtained from the registrants of a popular research company in Japan. A web-based self-administered questionnaire survey was conducted from November 20–22, 2017. We defined six strata by sex (men and women) and age (20–24, 25–29, and 30–34 years) to adopt a stratified sampling. Among the total of 319,913 registrants aged 20 to 34 years, the online survey recruiting was continued until fulfilling 1,250 participants in each stratum. The total of 7,500 participants completed answering the questionnaire. This research project focused on not only student loans but also on the other socio-economic factors; therefore, the data also included young adults who never enrolled in a university. In this study, we targeted current university students, graduates, and dropouts. We excluded 3,351 participants who had never enrolled in a university. Thus, the final analytic population was 4,149.

### Independent variable: student loans

The main independent variable was the types of student financial support. The information was obtained using the following modified question referring to an early Japanese survey^20^: “I will ask you about scholarships and student loans. Have you used scholarships or student loans when you were a university student?”. The possible answers were “Borrowed student loans (repayment required) from Japan Student Services Organization (former name: Japan Scholarship Foundation),” “Borrowed student loans (repayment required) from a university or a private sector,” “Used scholarships (repayment not required) from a university or a private sector,” “None”, and “I don’t know”. Then, we categorized the first and the second potential answer as “Student loans”, the third potential answer as “Scholarships”, choosing both the first or the second and the third potential answers as “Both types”, and none as “None”.

We also obtained the total amount of student loan debt among respondents with student loan or both types from the following question: “What was the total amount of student loan debt that you have borrowed?”. The possible answers were “0.5 million yen or less”, “0.5 to 1.0 million yen”, “1.0 to 2.0 million yen”, “2.0 to 3.0 million yen”, “3.0 to 4.0 million yen”, “4.0 to 5.0 million yen”, “5.0 to 6.0 million yen”, “6.0 to 7.0 million yen”, “7.0 to 8.0 million yen”, “8.0 to 9.0 million yen”, “9.0 to 10.0 million yen”, “10.0 million yen or more”, and “I don’t know”. We trichotomized the total amount of student loan debt based on the tertile of the number of respondents: <2 million yen, 2 to 4 million yen, and ≥4 million yen.

### Dependent variable: psychological distress

The main dependent variable is psychological distress measured using the Kessler Psychological Distress Scale (K6) score which has been used in the widely published studies.^21^^,^^22^ The K6 score ranges 0 (no distress) to 24 (maximum distress), and assesses non-specific psychological distress during the past 30 days.^21^ In the main analysis, we used a dichotomic variable of the K6 score using a cut-off point at 12/13, which is defined as having severe psychological distress.^23^ To verify the validity of the results from the main analysis, we also used the sum of the K6 score as a numeric variable.

### Covariates

We used these factors as covariates: Age (20–24, 25–29, and 30–34 years old), sex (man and woman), and current student status (current university student, and graduates or dropouts). We also included the following socioeconomic variables as covariates^13^: educational attainment (4-year university, 6-year university [only medical, dental, and pharmaceutical department], and master’s or doctorate’s degrees), sources of the enrolled university (public and private), father’s educational attainment (less than university, and university and higher), mother’s educational attainment (less than university, and university and higher), and current parents’ annual household income (high [≥6 million yen], middle [3 to 6 million yen], low [>0 to 3 million yen], and none [0 yen]).

### Statistical analysis

We conducted a Poisson regression analysis with a robust error variance to estimate prevalence ratios (PRs) and 95% confidence intervals (CIs) for the dichotomized dependent variable (having severe psychological distress and none).^24^ PRs can be interpreted as relative risks.^24^ To verify the results from the main analysis, we also conducted a multivariable linear regression analysis and estimated non-standardized coefficients (βs) and 95% CIs. Because there was a significant interaction between current student status and the status of borrowing student loans (the *P*-value of the additive interaction term was 0.18 in the Poisson regression model with a robust error variance, but the *P*-value of the interaction term was less than 0.01 in the multivariable linear regression model), we conducted stratified analyses by current student status. We created two models; in model 1, age and sex were adjusted. In model 2, we added educational attainment, sources of the enrolled university, father’s educational attainment, mother’s educational attainment, and current parents’ annual household income in model 1. We mainly focused on the associations of student loans with psychological distress, because sample sizes of scholarships and both types were too small (*n* = 94 and *n* = 74, respectively). Therefore, the association of the total amounts of student loan debt with severe psychological distress was examined among graduates and dropouts with student loans only. eTable 1 shows the information of missing values in each variable. Based on the assumption of missing at random, we conducted a single imputation using the k-nearest neighbor algorithm from the R package named “DMwR”.^25^ A *P*-value <0.05 (two-tailed) was considered statistically significant. The *P*-value of the additive interaction was calculated using the Excel spreadsheet provided in the early study,^26^ and the other analyses were conducted by R (ver. 3.5.0; R Foundation for Statistical Computing, Vienna, Austria) with R studio (ver. 1.0.153) on Macintosh OS.

### Ethics approval

This study was reviewed and approved by the ethics committee of the Tohoku University Graduate School of Dentistry (2017-3-6).

## RESULTS

The median age was 27 years old (the first and third quartiles were 23 and 31, respectively), and the percentage of women was 43.7% (*n* = 1,813). Graduates, dropouts, and current university students were 70.8% (*n* = 2,937), 5.6% (*n* = 233), and 23.6% (*n* = 979), respectively. The percentage of participants with student loans was 33.8% among graduates and 35.2% among current university students, respectively. About 90% of those with student loans had borrowed from the JASSO. Table 1 and Table 2 present the characteristics of participants stratified by current student status. The median category of the total amount of student loan debt was 2.0 to 3.0 million yen. Compared with participants without student loans and scholarships, participants with student loans had low parents’ educational attainment and low current parents’ annual household income.

**Table 1.** Characteristics of graduates and dropouts according to the types of student financial support

		Graduates and dropouts							
		(*n* = 3,170)							

		Types of student financial support							
		None		Student loans		Scholarships		Both types	
		(*n* = 1,826, 62.2%)		(*n* = 994, 33.8%)		(*n* = 66, 2.2%)		(*n* = 51, 1.7%)	

		*n*	%	*n*	%	*n*	%	*n*	%
Total amounts of student loan debt (million yen)	Low (>0 to <2.0)	—		208	26.8	—		10	23.3
	Middle (2.0 to 4.0)	—		380	49.0	—		18	41.9
	High (≥4.0)	—		187	24.1	—		15	34.9
Source of student loans	Japan Student Services Organization	—		889	89.4	—		47	92.2
	Others	—		105	10.6	—		4	7.8
**Covariates**									
Age, years	20–24	233	12.8	186	18.7	17	25.8	11	21.6
	25–29	740	40.5	437	44.0	23	34.8	26	51.0
	30–34	853	46.7	371	37.3	26	39.4	14	27.5
Sex	Women	1,017	55.7	524	52.7	42	63.6	28	54.9
	Men	809	44.3	470	47.3	24	36.4	23	45.1
Educational attainment	4-year university	1,630	89.3	852	85.7	51	77.3	37	72.5
	6-year university	42	2.3	18	1.8	1	1.5	1	2.0
	Master’s or doctorate’s degrees	154	8.4	124	12.5	14	21.2	13	25.5
Sources of the enrolled university	Public	365	21.8	249	28.6	15	28.8	9	23.7
	Private	1,307	78.2	621	71.4	37	71.2	29	76.3
Father’s educational attainment	Less than university	573	34.3	438	50.0	22	34.4	20	43.5
	University and higher	1,098	65.7	438	50.0	42	65.6	26	56.5
Mother’s educational attainment	Less than university	1,139	68.0	705	77.6	42	67.7	33	66.0
	University and higher	535	32.0	203	22.4	20	32.3	17	34.0
Current parents’ annual household income (million yen)	High (≥6.0)	378	34.3	156	24.3	13	29.5	8	22.2
	Middle (3.0 to 6.0)	441	40.0	261	40.7	20	45.5	16	44.4
	Low (>0 to 3.0)	251	22.8	207	32.2	8	18.2	11	30.6
	None (0)	33	3.0	18	2.8	3	6.8	1	2.8
**Dependent variable**									
Severe psychological distress, K6 score ≥13	Having severe psychological distress	214	11.7	146	14.7	7	10.6	7	13.7
Psychological distress (K6 score; mean and standard deviation)		5.3	5.7	6.0	5.9	6.1	5.6	6.0	5.8

**Table 2.** Characteristics of current university students according to the types of student financial support

		University students							
		(*n* = 979)							

		Types of student financial support							
		None		Student loans		Scholarships		Both types	
		(*n* = 548, 59.3%)		(*n* = 325, 35.2%)		(*n* = 28, 3.0%)		(*n* = 23, 2.5%)	

		*n*	(%)	*n*	(%)	*n*	(%)	*n*	(%)
Source of student loans	Japan Student Services Organization	—		302	92.9	—		21	91.3
	Others	—		23	7.1	—		2	8.7
**Covariates**									
Age, years	20–24	515	94.0	309	95.1	25	89.3	17	73.9
	25–29	26	4.7	14	4.3	3	10.7	6	26.1
	30–34	7	1.3	2	0.6	0	0.0	0	0.0
Sex	Women	331	60.4	184	56.6	17	60.7	10	43.5
	Men	217	39.6	141	43.4	11	39.3	13	56.5
Educational attainment	Four-year university	448	81.8	266	81.8	17	60.7	10	43.5
	Six-year university	40	7.3	9	2.8	4	14.3	4	17.4
	Master’s or doctorate’s degrees	60	10.9	50	15.4	7	25.0	9	39.1
Sources of the enrolled university	Public	178	36.5	97	35.3	6	28.6	3	21.4
	Private	310	63.5	178	64.7	15	71.4	11	78.6
Father’s educational attainment	Less than university	148	29.2	154	52.7	4	16.0	8	36.4
	University and higher	358	70.8	138	47.3	21	84.0	14	63.6
Mother’s educational attainment	Less than university	324	62.7	243	79.4	15	57.7	17	73.9
	University and higher	193	37.3	63	20.6	11	42.3	6	26.1
Current parents’ annual household income (million yen)	High (≥6.0)	158	50.8	80	40.8	8	50.0	2	11.1
	Middle (3.0 to 6.0)	71	22.8	66	33.7	4	25.0	8	44.4
	Low (>0 to 3.0)	68	21.9	49	25.0	4	25.0	8	44.4
	None (0)	14	4.5	1	0.5	0	0.0	0	0.0
**Dependent variable**									
Severe psychological distress, K6 score ≥13	Having severe psychological distress	59	10.8	32	9.8	2	7.1	3	13.0
Psychological distress (K6 score; mean and standard deviation)		5.6	5.5	5.3	5.1	5.4	4.9	6.1	7.0

Table 3 shows the association of the types of student financial support with psychological distress stratified by current student status after imputation. After adjusting for age and sex, there were significant negative associations between student loans and severe psychological distress among graduated and dropouts (PR 1.22; 95% CI, 1.01–1.47). There were no significant associations of scholarships and both types with severe psychological distress (PR of scholarship, 0.84; 95% CI, 0.41–1.70 and PR of both types, 1.10; 95% CI, 0.55–2.22). In the fully adjusted model, graduates and dropouts with student loans had a high possibility of having severe psychological distress compared with ones without student loans and scholarships (PR 1.26; 95% CI, 1.04–1.53). The total amount of student loan debt was also associated with a high possibility of having severe psychological distress compared with ones without student loan debt (PR of <2 million yen, 1.04; 95% CI, 0.77–1.41, PR of 2 to 4 million yen, 1.26; 95% CI, 0.96–1.65, and PR of ≥4 million yen, 1.44; 95% CI, 1.02–2.03). Current university students with student loans had a low possibility of having severe psychological distress (PR 0.91; 95% CI, 0.60–1.37), compared with ones without student loans and scholarships after adjusting for covariates. Table 4 presents the results from the multivariable linear regression models. The models also show that the total amount of student loan debt was associated with a high psychological distress score compared with ones without student loans and scholarships (β of <2 million yen, 0.04; 95% CI, −0.65 to 0.74, β of 2 to 4 million yen, 0.82; 95% CI, 0.17–1.47, and β of ≥4 million yen, 1.02; 95% CI, 0.14–1.90). eTable 2 presents the results from models with pairwise deletion, and these were consistent with the results after imputation.

**Table 3.** Associations of the types of student financial support and the total amount of student loan debt with severe psychological distress^a^ from Poisson regression models with a robust error variance stratified by current student status after imputation

			Model 1^b^		Model 2^c^	

			PR	95% CI	PR	95% CI
**Among graduates and dropouts**			(*n* = 3,170)		(*n* = 3,170)	
Types of student financial support		None	Reference		Reference	
		Student loans	1.22	1.01, 1.47	1.26	1.04, 1.53
		Scholarships	0.84	0.41, 1.70	0.87	0.43, 1.77
		Both types	1.10	0.55, 2.22	1.18	0.59, 2.39

	**Among graduates and dropouts with only student loan and none**		(*n* = 3,053)		(*n* = 3,053)	
	Total amounts of student loan debt	None	Reference		Reference	
		<2.0 million yen	1.02	0.76, 1.39	1.04	0.77, 1.41
		2.0 to 4.0 million yen	1.21	0.93, 1.58	1.26	0.96, 1.65
		≥4.0 million yen	1.38	0.98, 1.94	1.44	1.02, 2.03

**Current university students**			(*n* = 979)		(*n* = 979)	
Types of student financial support		None	Reference		Reference	
		Student loans	0.97	0.65, 1.44	0.91	0.60, 1.37
		Scholarships	0.66	0.17, 2.58	0.72	0.18, 2.88
		Both types	1.09	0.35, 3.42	1.12	0.36, 3.52

CI, confidence interval; PR, prevalence ratio.

^a^Severe psychological distress was assessed by the Kessler Psychological Distress Scale by a cut-off point at 12/13.

^b^Model 1: Age and sex were adjusted.

^c^Model 2: Model 1 + educational attainment, sources of the enrolled university, father’s educational attainment, mother’s educational attainment, and current parents’ annual household income were adjusted.

The single imputation was conducted using types of student financial support, amounts of student loan debt, current student status, age, sex, educational attainment, sources of the enrolled university, father’s educational attainment, mother’s educational attainment, current parents’ annual household income, and the K6 score by the the k-nearest neighbor algorithm from the R package named “DMwR.”

**Table 4.** Associations of the types of student financial support and the total amount of student loan debt with psychological distress^a^ from linear regression models stratified by current student status after imputation

			Model 1^b^		Model 2^c^	

			β	95% CI	β	95% CI
**Among graduates and dropouts**			(*n* = 3,170)		(*n* = 3,170)	
Types of student financial support		None	Reference		Reference	
		Student loans	0.61	0.17, 1.06	0.62	0.17, 1.07
		Scholarships	0.59	−0.85, 2.02	0.64	−0.80, 2.07
		Both types	0.57	−1.05, 2.20	0.60	−1.03, 2.23

	**Among graduates and dropouts with only student loan and none**		(*n* = 3,053)		(*n* = 3,053)	
	Total amounts of student loan debt	None	Reference		Reference	
		<2.0 million yen	0.08	−0.60, 0.77	0.04	−0.65, 0.74
		2.0 to 4.0 million yen	0.79	0.16, 1.43	0.82	0.17, 1.47
		≥4.0 million yen	0.98	0.11, 1.86	1.02	0.14, 1.90

**Current university students**			(*n* = 979)		(*n* = 979)	
Types of student financial support		None	Reference		Reference	
		Student loans	−0.35	−1.09, 0.38	−0.57	−1.34, 0.19
		Scholarships	−0.42	−2.48, 1.65	−0.39	−2.46, 1.68
		Both types	−0.19	−2.48, 2.10	−0.48	−2.80, 1.84

β, non-standardized coefficient; CI, confidence interval.

^a^Psychological distress was assessed by the Kessler Psychological Distress Scale.

^b^Model 1: Age and sex were adjusted.

^c^Model 2: Model 1 + educational attainment, sources of the enrolled university, father’s educational attainment, mother’s educational attainment, and current parents’ annual household income were adjusted.

The single imputation was conducted using types of student financial support, amounts of student loan debt, current student status, age, sex, educational attainment, sources of the enrolled university, father’s educational attainment, mother’s educational attainment, current parents’ annual household income, and the K6 score by the the k-nearest neighbor algorithm from the R package named “DMwR.”

## DISCUSSIONS

This is the first study to report the associations of student loans with psychological distress in Japan. While graduates and dropouts with student loans had a high possibility of having severe psychological distress compared with ones without student loans and scholarships, there was no significant association among current university students. There were also significant associations of the total amount of student loan debt with psychological distress among graduates and dropouts.

The associations of borrowing student loans and the total amount of student loan debt with psychological distress were relatively moderate. However, the burden of student loan debt on the population health can be considerable due to the high rate of borrowing student loans. The inflation of the value of educational credentials has been proceeding in Japan because global economic competition demands highly skilled workers.^27^ More people desire higher education to get high-skill occupations.^27^ Indeed, among the total population who graduated from high school, the rate of university enrolments has been increasing to 52.6% in 2017 from 24.7% in 1989.^28^ Thence, about 40% of them borrowed student loans in 2017.^17^^,^^18^ We should pay attention to the associations between student loan debt and health.

The current results are inconsistent with the previous study in the United States.^13^ The current results show that there was no significant association among current university students, but graduates and dropouts with student loans had a high possibility of severe psychological distress. Walsemann and colleagues reported that the significant associations of student loans with poor mental health were observed among both current students and graduated adults.^13^ This might depend on different situations among university students between Japan and the United States. In the United States, parents paid just 34% in the tertiary educational resources, and most students needed to pay from their income, grants, and loans.^29^ Therefore, American students can have recognized the burden of student loan repayment well. On the other hand, most Japanese undergraduate students mainly relied on parents’ monetary support (over 60% of the total amount of living expense),^18^ and half of the Japanese students did not recognize the burden of student loan repayment.^30^ Besides, students with student loans obtained the monthly payment. Therefore, they might not worry about the economic circumstances when they were students. Indeed, there was the significant interaction between current student status and the status of borrowing student loans. This result means that the association between student loans and psychological distress was weak among current university student compared with ones among graduates and dropouts. However, student loan debt can be a burden after graduation. From the current results, the mean annual household income of graduates and dropouts was 3.0 to 4.0 million yen. Although the monthly student loan repayment is relatively small compared with the mean annual household income, the repayment to the JASSO is fixed amount regardless of their annual income.^16^ We observed dose-response associations between student loan debt and psychological distress. A higher amount of student loan debt links to a higher monthly student loan repayment and a longer period.^16^ The long-term monthly student loan repayment might place a financial burden on household income and bring repayment worry and stress.

It is difficult to interpret the results of the associations of scholarships and using both types with psychological distress, due to the small sample size. As earlier reports indicated, there are limitations to use scholarships for university students in Japan.^11^^,^^16^^,^^17^ Students with scholarships might be in a specific situation; therefore, unobserved confounders still might exist. Further research should focus on students with scholarships.

This study has some limitations. First, the data was obtained from the web-based self-administered questionnaires. The previous study indicated that respondents who were recruited in a web survey tended to be younger compared with the Japanese population.^31^ In addition, there is a possibility of misclassification of the total amount of student loan debt due to the use of non-validated question. Some participants might answer the current amount of student loan debt at the survey, instead of the accumulated amount of student loan debt when participants graduated or dropped out. However, the percentage of borrowing student loans and the total amount of student loan debt are relatively consistent with earlier reports.^17^^,^^18^ Second, this study design was a cross-sectional; therefore, the data did not include health status when participants enrolled in university. Although we adjusted for parents’ socioeconomic status, unobserved variables might confound the associations. In the future, a cohort study is needed.

### Conclusions

There was a significant cross-sectional association between student loans and psychological distress. Although scholarships have been offered since 2018 by JASSO, the number of students who use scholarships is still few.^16^ To alleviate the burden of the student loan debt repayment, reform of the current system is still desired, including broadening the application of student grant aid and adjusting the amount of debt repayments based on their income.^16^ Policymakers should note the cross-sectional associations of student loan debt with psychological distress.

## APPENDIX A. SUPPLEMENTARY DATA

eTable 1. Description of missing values

eTable 2. Associations of the types of student financial support and the total amount of student loan debt with psychological distress^a^ from Poisson regression models with a robust error variance using pairwise deletion stratified by current student status
