# Supplementary material for: Student Loans and Psychological Distress: A Cross-sectional Study of Young Adults in Japan
Source: J Epidemiol. 2020 Oct 5;30(10):436–41. doi: 10.2188/jea.JE20190057 (PMC7492703; doi:10.2188/jea.JE20190057)
Supplement: Supplementary file 1 [file je-30-436-s001.pdf]

eTable 1. Description of missing values

Variables	Number of missing (%)		Types of variable in the imputation (Categorical or continuous variables)
Types of student financial support	288	(6.9)	A categorical variable
Total amounts of student loan debt	589	(14.2)	A categorical variable
Current student status	0	(0)	A categorical variable
Age	0	(0)	A categorical variable
Sex	0	(0)	A categorical variable
Educational attainment	0	(0)	A categorical variable
Sources of the enrolled university	0	(0)	A categorical variable
Father's educational attainment	447	(10.8)	A categorical variable
Mother's educational attainment	392	(9.4)	A categorical variable
Current parents' annual household income	1,653	(39.8)	A categorical variable
K6 score	0	(0)	A categorical variable

We used a weighted average of observed values of the identified neighbors, and the number of neighbors was defined as 5 in the imputation.

eTable 2. Associations of the types of student financial support and the total amount of student loan debt with psychological distress[a] from Poisson regression models with a robust error variance using pairwise deletion stratified by current student status

		Model 1[b]		Model 2[c]	
		PR	95% CI	PR	95% CI
**Among graduates and dropouts**		(n=2,937)		(n=1,481)	
Types of student financial support	None	Reference		Reference	
	Student loans	1.23	1.01, 1.50	1.42	1.08, 1.88
	Scholarships	0.86	0.42, 1.75	0.92	0.36, 2.36
	Both types	1.13	0.56, 2.28	1.71	0.76, 3.82
**Among graduates and dropouts with only student loan and none**		(n=2,601)		(n=1,364)	
Total amounts of student loan debt	None	Reference		Reference	
	<2.0 million yen	1.17	0.81, 1.68	1.19	0.73, 1.94
	2.0 to 4.0 million yen	1.27	0.97, 1.66	1.41	0.98, 2.03
	≥4.0 million yen	1.44	1.02, 2.03	1.68	1.09, 2.60
**Current university students**		(n=924)		(n=429)	
Types of student financial support	None	Reference		Reference	
	Student loans	0.91	0.61, 1.37	0.85	0.47, 1.51
	Scholarships	0.64	0.16, 2.52	1.80	0.45, 7.20
	Both types	1.04	0.33, 3.34	2.47	0.84, 7.26

CI, confidence interval; PR, prevalence ratio.

[a]Severe psychological distress was assessed using the Kessler Psychological Distress Scale with a cut-off point at 12/13.

[b]Model 1: Age and sex were adjusted.

[c]Model 2: Model 1 + educational attainment, sources of the enrolled university, father's educational attainment, mother's educational attainment, and current parents' annual household income were adjusted.
